# CARE 2.0: reducing false-positive sequencing error corrections using machine learning

**DOI:** 10.1186/s12859-022-04754-3

**Published:** 2022-06-13

**Authors:** Felix Kallenborn, Julian Cascitti, Bertil Schmidt

**Affiliations:** Department of Computer Science, Johannes Gutenberg University Mainz, Mainz, Germany

**Keywords:** Next-generation sequencing, Error correction, Machine learning

## Abstract

**Background:**

Next-generation sequencing pipelines often perform error correction as a preprocessing step to obtain cleaned input data. State-of-the-art error correction programs are able to reliably detect and correct the majority of sequencing errors. However, they also introduce new errors by making false-positive corrections. These correction mistakes can have negative impact on downstream analysis, such as *k*-mer statistics, de-novo assembly, and variant calling. This motivates the need for more precise error correction tools.

**Results:**

We present CARE 2.0, a context-aware read error correction tool based on multiple sequence alignment targeting Illumina datasets. In addition to a number of newly introduced optimizations its most significant change is the replacement of CARE 1.0’s hand-crafted correction conditions with a novel classifier based on random decision forests trained on Illumina data. This results in up to two orders-of-magnitude fewer false-positive corrections compared to other state-of-the-art error correction software. At the same time, CARE 2.0 is able to achieve high numbers of true-positive corrections comparable to its competitors. On a simulated full human dataset with 914M reads CARE 2.0 generates only 1.2M false positives (FPs) (and 801.4M true positives (TPs)) at a highly competitive runtime while the best corrections achieved by other state-of-the-art tools contain at least 3.9M FPs and at most 814.5M TPs. Better de-novo assembly and improved *k*-mer analysis show the applicability of CARE 2.0 to real-world data.

**Conclusion:**

False-positive corrections can negatively influence down-stream analysis. The precision of CARE 2.0 greatly reduces the number of those corrections compared to other state-of-the-art programs including BFC, Karect, Musket, Bcool, SGA, and Lighter. Thus, higher-quality datasets are produced which improve *k*-mer analysis and de-novo assembly in real-world datasets which demonstrates the applicability of machine learning techniques in the context of sequencing read error correction. CARE 2.0 is written in C++/CUDA for Linux systems and can be run on the CPU as well as on CUDA-enabled GPUs. It is available at https://github.com/fkallen/CARE.

**Supplementary Information:**

The online version contains supplementary material available at 10.1186/s12859-022-04754-3.

## Background

Modern sequencing technologies can produce high-coverage datasets consisting of many millions or even billions of short sequencing reads. Produced reads, however, are not perfect but are affected by noise which manifests in the form of sequencing errors. Such errors can affect down-stream analysis in a negative way. Error correction software is often employed to remove many of these sequencing errors making it an important building block in next-generation sequencing (NGS) processing pipelines including genome assembly [1] and SNP calling [2].

Current state-of-the-art error correctors are often classified by their underlying algorithmic approach into *k*-mer based (1) and MSA-based (2) methods.

1. In a *k*-mer based approach the *k*-mer spectrum of a collection of sequencing reads is inspected to identify *k*-mers which are error-free with high confidence, so-called *solid* *k*-mers. *k*-mers which are not solid are called *weak*. Often, *k*-mers are distinguished as solid or weak based on their frequency in the dataset given a supplied frequency threshold. *k*-mers which reach the threshold are considered solid. *k*-mer based error correction algorithms typically try to replace weak *k*-mers by similar solid *k*-mers. While this approach is simple and fast, it usually suffers from a great number of false-positive (FP) corrections because low frequency correct, but weak, *k*-mers may be changed into erroneous, but solid *k*-mers which appear more often. Due to its simplicity, this approach is used in many error correction tools such as SGA-EC [3], Musket [4], RACER [5], Lighter [6], Blue [7], BFC [8], BLESS [9], and RECKONER [10].
2. MSA-based algorithms identify groups of similar sequences and arrange them in a multiple sequence alignment (MSA). In contrast to changing individual *k*-mers in isolation, MSA-based error correction utilizes the additional information contained in the MSA, such as per-column coverage, and sequence contents of positions surrounding a potentially erroneous position. This typically allows for higher error correction precision. However, a major drawback of the MSA-based approach is its high computational complexity to construct (approximate) MSAs. The first MSA-based error correctors specifically designed for Illumina data were Coral [11] and ECHO [12]. More recent examples of alignment-based error correctors are Fiona [13], Karect [14], Bcool [15], BrownieCorrector [16], and CARE [17]. These tools can be further distinguished by their utilized data structures (such as suffix trees/arrays, hash tables, or de Bruijn graphs) and their detailed correction heuristics/policies.

CARE is an MSA-based error correction tool which performs highly accurate corrections, with up to two orders-of-magnitude smaller FP-rate on average compared to other state-of-the-art programs. Furthermore, CARE is able to utilize CUDA-enabled GPUs to speed up its computation and reduce the high runtime associated with MSA construction. In this paper, we introduce CARE 2.0—an extension of CARE. The new contributions are three-fold.

First, CARE 2.0 includes a dedicated code path for paired-end reads. Previously, all reads were treated as unpaired. Second, we incorporate machine learning techniques in the form of random forests into the correction algorithm to further increase both precision and sensitivity. The random forests hereby replace our hand-crafted conditions to decide whether a specific position in a read should be modified. This is in contrast to previous recent machine learning approaches like Athena [18] and Lerna [19] which try to find optimal input parameters for existing correction algorithms. Third, the algorithm has been optimized to reduce both runtime and memory consumption on both CPUs and GPUs.

We confirm the benefits of the new approach using simulated as well as real-world Illumina data in down-stream analysis. Our experiments show that on average CARE 2.0 reduces the number of FPs by a factor of 1.8 compared to CARE while achieving a similar number of TPs on simulated datasets thereby vastly outperforming other state-of-the-art tools in terms of FP-rate such as Musket, SGA, Karect, Bcool, Lighter, and BFC. Additionally, the de-novo assembly quality as well as the *k*-mer spectra of corrected real-world datasets are improved.

## Implementation

### Workflow

The algorithm of CARE 2.0 can be split into three separate phases: *Construction*, *Correction*, and *Merge*.

#### Construction phase

In the construction phase each sequencing read is inserted into multiple hash tables. For each read $r_i$ *h* hash values, which form a so-called read signature *S*, are computed where *h* denotes the number of hash tables and used hash functions. Each hash function is applied to every canonical *k*-mer of $r_i$. The read signature then consists of the smallest observed hash value of each hash function. Finally, the key-value pair (*S*[*l*], *i*) is inserted into the *l*-th hash table.

#### Correction phase

**Fig. 1:**
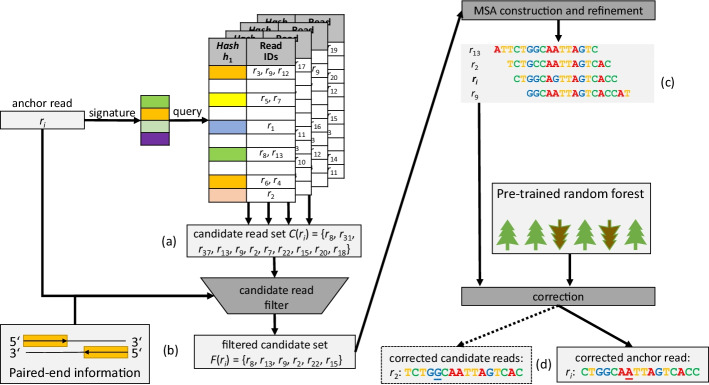
Workflow of CARE 2.0: **a** The signature of an anchor read ($r_i$) is determined by minhashing and used to query the precomputed hash tables. The retrieved reads form the candidate read set $C(r_i)$. **b** All reads in $C(r_i)$ are aligned to $r_i$. Reads with a relatively low semi-global pairwise alignment quality are removed, resulting in the filtered set of candidate reads ($F(r_i)$). **c** The initial MSA is constructed around the center $r_i$ using $F(r_i)$. The MSA is refined by removing candidate reads with a significantly different pattern from the anchor (i.e. $r_{15}, r_{22}, r_7$ in the example). **d** The anchor read (the seventh nucleotide in $r_i$ in the example) and optionally some of the candidates are corrected (the fifth nucleotide in $r_2$ in the example), using a provided random forest trained for correction

Figure 1 illustrates the general workflow for correction of read $r_i$ in CARE 2.0. First, the read signature *S* is computed again which is then used to query the collection of hash tables. The result of this operation is a set of candidate reads *C* which can be used for correction of the anchor read $r_i$.

Subsequently, each read in *C* is aligned to the anchor read. In general, this means computing a semi-global alignment between anchor and candidate. Since we target Illumina reads the dominant type of sequencing errors are mismatches. Gaps within a sequence are thus not considered and only semi-global alignments without inner gaps are computed. This is implemented as an efficient shifted hamming distance calculation using bit-wise operations [20].

Next, a filter is applied to the set of candidates to remove reads which do not provide sufficient information for the correction process. A perfect filter would only keep candidates which originate from the same genomic location as the anchor read. In CARE 2.0, the filter inspects the alignments and discards candidates whose alignment contains many mismatches, or does not sufficiently overlap the anchor read. Specifically, the ratio between number of mismatches and overlap length is considered. If the ratio surpasses a threshold the corresponding candidate is removed from the candidate set. Candidates which pass the filter are assumed to be similar to the anchor.

Let $F(r_i)$ denote the set of candidate reads which passed the filter. The next step is the construction of an MSA *M* containing all candidates in $F(r_i)$ together with anchor read $r_i$. This is achieved using the previously calculated pair-wise alignments between anchor and candidates in a manner similar to the well known STAR approximation algorithm [21]. In the MSA, we store nucleotide counts and sum of nucleotide weights per column per possible nucleotide (A,C,G,T). Candidates with a better alignment have higher nucleotide weights. If sequence quality scores are used, better quality scores also lead to higher weights. The *consensus* of a column is defined as the nucleotide with the greatest nucleotide weight. The *support* of a column is the relative weight of the consensus nucleotide.

After construction, *M* could already be used to apply corrections to anchor $$r_i$$ and achieve reasonably good results. However, this can be inaccurate for anchors that can be mapped to (inexact) repeat regions or anchors with multiple errors. Thus, we apply a second filter labeled *MSA refinement*, which attempts to remove candidates from both $$F(r_i)$$ and *M* which originate from a different genomic region. This is done by inspecting the columns of *M* to find candidates with different patterns than the anchor read. Let column *e* be a column which is covered by the anchor read. The column contents are inspected to find a nucleotide *X* which is different from the column consensus with a frequency exceeding a certain threshold (default: $$0.3 \times estimated dataset coverage$$). If *X* is equal to the corresponding base in the anchor read, candidates which do not contain *X* at their respective position in column *e* are removed from both *M* and $$F(r_i)$$. Otherwise, candidates will be removed which match *X* at the corresponding position. This process of column inspection and removal is repeated at most five times. After each iteration, the MSA consensus is recalculated. MSA refinement will stop early as soon as no candidate is removed during an iteration.

The refined MSA will be used for error correction. Error correction is performed by changing nucleotides in the anchor to the corresponding column consensus of the MSA. We distinguish two cases for anchor correction based on the quality of the MSA. For high-quality MSAs, each position of the anchor is changed to the consensus unconditionally. On the other hand, only positions with high confidence in the MSA are changed in the case of a low-quality MSA. A position with high confidence has a support value greater than 0.90 in the corresponding column, and the nucleotide of the anchor for this column does appear at most twice in the column. To classify an MSA as either high-quality or low-quality, column support and column coverage are aggregated to find the minimum column support, average column support, and minimum column coverage. Subsequently, thresholds are applied to the computed values to distinguish high-quality MSAs from low-quality MSAs.

High-quality MSAs do not only provide reliable information about columns which correspond to positions in the anchor but can also extend to the left and to the right of the anchor, respectively. The column coverage decreases towards the left end and right end. This also means that columns which are close to the anchor read may have decent coverage, and can be used to correct the candidates of the current anchor. Let [*b*, *e*) be the column interval which is covered by the anchor read. CARE 2.0 will produce a candidate correction if the candidate is fully contained in the column range $$[b - x, e+x)$$, where $$x = 15$$ is the default value. The same read can be corrected as a candidate in MSAs of different anchors.

#### Merge phase

After correction, all results are sorted and corrections are grouped by read in order to merge each read’s corrections into a final corrected read, which is subsequently written to the result file. For each read, there exists an anchor correction and zero or more possible candidate corrections. If less than three candidate corrections are present, the anchor correction is treated as the final correction result. Otherwise, it is only used if it is equal to all of its candidate corrections. Otherwise, the original read remains unmodified, i.e. no correction is performed, and the original read is written to file.

### Novel features of CARE 2.0

CARE 2.0 provides alternatives for two components of the workflow. First, the candidate filter is improved to be able to utilize the inherent information contained in paired-end reads. Second, a pre-trained Random Forest classifier can be utilized to determine which positions of a read should be modified during the correction step. Our results show that these two modifications are able to greatly reduce the number of false-positive corrections while maintaining a high number of true positives. Additionally, using Random Forests to aid error correction can further increase true-positive corrections. It is possible to opt out of the CARE 2.0 alternatives, in which case the program behaves equivalently to CARE 1.0, but with improved resource usage.

#### Paired-end filter

Paired-end sequencing technologies produce pairs of reads. The two reads in a read pair come from the same genomic region, and are usually separated by only a few hundred base pairs. For any given read in a read pair, we will refer to the second read of that pair as its *mate*.

In CARE 2.0, both reads of a pair can be processed simultaneously. After finding the two candidate sets and computing the respective pair-wise alignments with the two anchors, candidates will be filtered to remove candidates of different regions as explained above. Here, the pair information can play an important role. The two candidate sets are inspected to find candidates of the same read pair, where one of the reads is a candidate of one anchor, and the mate of that candidate is a candidate of the anchor’s mate. Such pairs of candidates are kept in the candidate sets unconditionally. We assume that this condition holds true only if the anchor read pair and the candidate read pair both originate from the same genomic region. The other candidates are filtered by the ratio between number of mismatches and size of overlap, which needs to be less than a threshold $t_{paired}$ (default: 0.06). For example, assume candidates of both an anchor read $a_0$ and its mate $a_1$ should be filtered. Let the candidate sets of $a_0$ and $a_1$ be $C(a_0)=\{r_0, r_5, r_{11}\}$ and $C(a_1)=\{r_4, r_8, r_{14}\}$, respectively. Two consecutive reads $r_{2*i}, r_{2*i+1},i \in {\mathbb {N}}$ form a read pair. Then $r_4$ and $r_5$ always pass the filter, because they originate from the same read pair. The remaining candidates are kept depending on their alignment quality to the corresponding anchor read.

#### Random forest-based correction

In general, Random Forests are an ensemble learning method [22] generating a collection of decision trees based on the available training data by separating instances from two predefined and known classes (positive and negative class) according to a number of features describing each instance. During the prediction process, i.e. classification of an instance of unknown class, each individual decision tree is applied to the instance’s features, yielding a probability for each class. The instance is then classified according to its average class probabilities over the set of decision trees. A final class label is assigned by comparing the instance’s positive class probability to a predefined threshold.

**Fig. 2:**
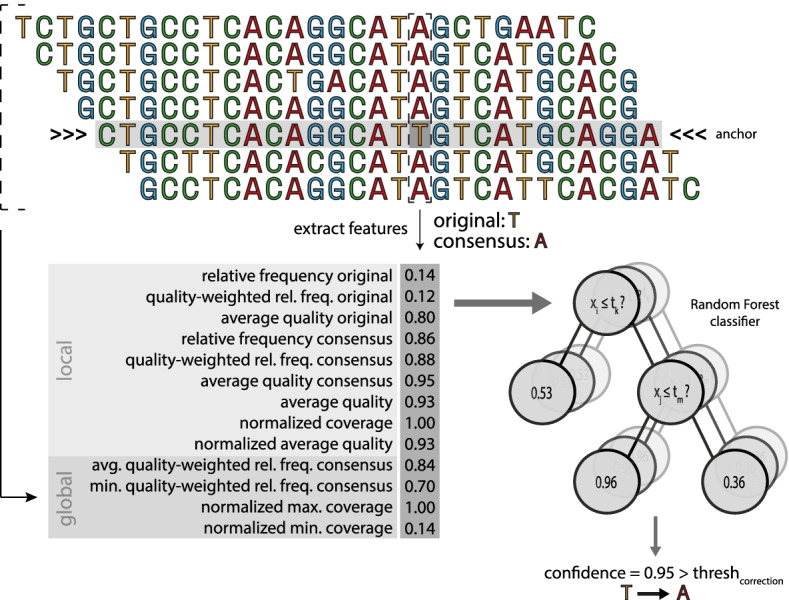
Example features for the dashed column in the MSA constructed for the anchor. The random forest will use the features to decide if anchor nucleotide **T** is changed into consensus nucleotide **A**. Quality scores / weights are not displayed for brevity

CARE 2.0 offers a Random Forest-based correction mode which differs in its approach to low-quality MSAs by utilizing a set of two distinct Random Forest classifiers to identify nucleotides to be corrected. As in the default mode, nucleotides of anchors of high-quality MSAs are replaced with the alignment consensus unconditionally, whereas for low-quality MSAs, we extract for each considered nucleotide position a variety of *features* from its MSA. Utilizing a Random Forest classifier, trained on simulated sequencing reads of a variety of genomes, we determine a confidence value for the correction of each nucleotide of the read based on its extracted features. Nucleotides whose correction confidence surpasses a user-defined correction threshold are replaced with the position’s MSA consensus. The choice of the threshold is a trade-off between sensitivity and precision. Lower thresholds will increase the number of corrected errors at the cost of an increase in false positives. Extracted features include both local features, limited to the MSA column of the considered read position, and global features, which take into account information from all columns of the read’s MSA. Local features include the relative frequencies of the original and consensus nucleotides, their respective sequencing qualities and the local coverage, while global features include arithmetic transformations of coverage and consensus frequency values over the length of the MSA. Figure 2 shows an example MSA with a selection of corresponding features. A separate Random Forest classifier and feature extraction are utilized in the case of candidate corrections, in which additional features such as the overlap of candidate and anchor are taken into account.

#### Performance improvements and additional features

CARE 2.0 includes a number of performance optimizations which improve both runtime and memory usage.

- CARE 1.0 requires an initial scan of the input file to find the number of reads and maximum read length to calculate the required size of a contiguous memory location large enough to store the reads. Reads are then stored to this memory in a second pass. In CARE 2.0, reads are loaded in chunks in a single pass, and are subsequently rearranged in memory.
- The memory footprint of hash tables has been reduced. This allows for using more hash tables to improve correction quality when available RAM is a limiting factor.
- Construction of the output file is faster. If possible, the GPU is used to sort the results to group results belonging to the same input read. The number of accesses to the hard-drive has been decreased in the case when correction results have been spilled to hard-drive because no more system memory was available. Finally, multiple cpu threads are used to overlap reading the input file, combining the original read with its correction result, and writing the output file.
- The usage of read quality scores usually improves correction quality. However, standard quality scores in 8-bit ASCII format require four times more memory compared to 2-bit encoded DNA sequences. CARE 2.0 offers an optional lossy binned representation of quality scores using only one bit or two bits per character, respectively. This allows for a more fine-grained trade-off between correction quality and memory usage.

### Program information

CARE 2.0 is written in C++17 and targets Linux workstations. It uses multi-threading to achieve parallelism on suitable hardware. CARE 2.0 comes in two versions, a CPU version, and a GPU version. The latter requires a system with one or more Nvidia GPUs compatible with CUDA 11. The GPU version allows for additional parallelism of the algorithm by moving the majority of computations to the GPU. CARE 2.0 provides additional python scripts to train other forests. Detailed instructions are included in the software repository.

## Results

CARE 2.0 has been evaluated on both simulated and real-world Illumina data. Table 1 lists the different datasets. Real-world datasets are publicly available. Simulated datasets have been generated using the ART read simulator with its built-in HiSeq 2000 profile. This produces reads with an error-rate of approximately one percent. Its error model is explained in [23]. The utilized program arguments for ART are available in the supplement. The correction quality is evaluated in terms of three categories. For simulated datasets, error-free versions of the datasets are also available from ART. Those are used to determine per-base counts of true-positive corrections (TP), false-positive corrections (FP), and related metrics.

**Table 1:** List of simulated datasets (S1–S4) and real-world datasets (R1–R3)

Name	Organism	Coverage	Reads
S1	C.elegans	30x	30.1M
S2	D.melanogaster	30x	36.0M
S3	Hum. Chr. 14	30x	26.5M
S4	Human	30x	914.7M
R1	C.elegans	58x	57.7M
R2	D.melanogaster	64x	75.9M
R3	Hum. Chr. 14	35x	36.5M

Simulated reads have length 100. Real reads have length 101. Accession numbers for R1 and R2 are SRR543736, and SRR988075, respectively. R3 is taken from GAGE Genome Assembly Gold-standard Evaluations

Let *u*, *e*, *c* be the nucleotide in the same read at the same position in the uncorrected file, error-free file, and corrected file, respectively. It is a true positive correction if $u \ne e \wedge e = c$. False positives are defined as $u = e \wedge e \ne c$.

The evaluation of real-world datasets is performed by comparing genome assemblies computed from uncorrected and corrected datasets. Assembly is performed with SPAdes v3.13.1 [24]. The assembly metrics which are used for comparison are obtained using QUAST v5.0.2 [25]. As a second type of real-world evaluation, *k*-mer spectra of corrected datasets are compared. Jellyfish v2.3.0 [26] is used to count low-coverage lost true *k*-mers, i.e. *k*-mers which are present in both the dataset and the corresponding reference genome, and which are missing in the corrected dataset. The results of CARE 2.0 are compared to other state-of-the-art error correction tools Musket v1.1, SGA v0.10.15, Karect (Github commit from 16th March 2015), Bcool (Github commit from 29th November 2018), Lighter v1.1.2, and BFC r181, as well as CARE 1.0 to confirm algorithmic improvements.

CARE 2.0 has the ability to use paired-end information of reads, and can perform error correction using a random forest. Unless stated otherwise, the benchmarks were conducted using the following program settings for CARE 1.0 and CARE 2.0. The *k*-mer size is set to 20. 48 hash tables are used. Standard 8-bit quality scores and candidate correction are enabled. Additionally, CARE 2.0 uses both the random forest for anchor correction and candidate correction, and the paired-end mode, using $t_{paired} = 0.06$ (default) for paired-end filtering. Tools other than CARE and CARE 2.0 are run with default settings. The exact program arguments for each tool are listed in the supplement.

### Training of random forests

We generated multiple simulated read datasets (each with read length 100 and 30x coverage) for the following reference genomes: C.elegans, D.melanogaster, Human Chr.14, Human Chr.15, Mus musculus Chr. 15, and A.thaliana. These datasets were used to train multiple Random Forest classifiers, consisting of 128 decision trees each, following a genome-wise leave-one-out approach, i.e. training only on data from at most 5 out of 6 reference genomes. The following evaluations were then performed using only those classifiers not trained on the genomes corresponding to the respective corrected datasets. That is, training data of C.elegans is not included in the forest to correct *S*1 and *R*1. D.melanogaster is excluded for correction of *S*2 and *R*2. Finally, the forest which was used to correct *S*3, *S*4, and *R*3, was not trained using reads from Human Chr.14 or Human Chr.15. All classifiers were trained using the scikit-learn toolkit [27] for Python 3 using the library’s default hyperparameters and converted to our own binary data format for use with CARE 2.0.

### Simulated data

**Fig. 3:**
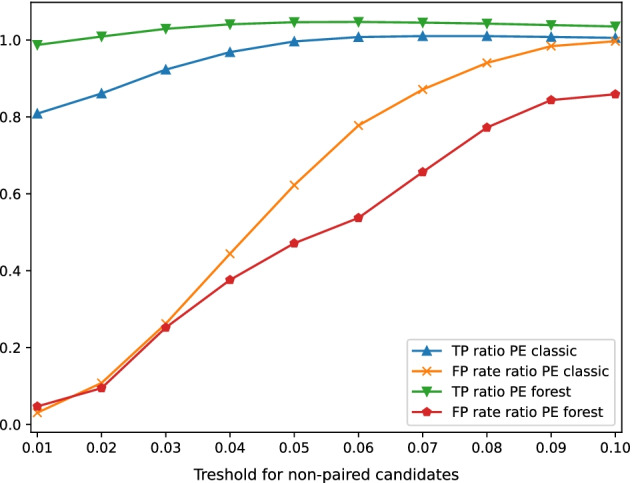
Comparison of CARE 2.0 paired-end mode to CARE 1.0, i.e single-end classic mode, on dataset S3. Results are normalized to CARE 1.0. For TP ratio greater numbers are better. For FP rate ratio, smaller numbers are better. FP rate is the rate of false-positive corrections in one million corrections

Each simulated dataset has been corrected by each tool. We performed a three-way comparison between original datasets, error-free datasets, and corrected datasets to obtain the number of TP and FP, as well as the number of false-positive corrections in one million corrections, i.e $10^6*FP/(FP+TP)$. Figure 3 shows a comparison between CARE 1.0 and CARE 2.0 to highlight the impact of the choice of threshold $t_{paired}$ on TPs and FPs. With the default value of 0.06, CARE 2.0 with random forest is able to reduce the number of false positives by a factor of 2 (ratio 0.5), and to slightly increase the number of true corrections.

**Fig. 4:**
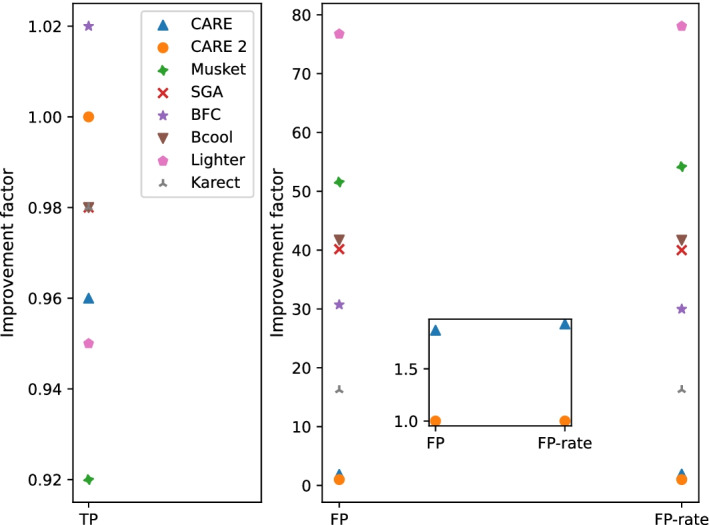
Average improvement factors of CARE 2.0. For true-positive corrections (TP) greater numbers are better. For false-positive corrections (FP) and false-positive rate per million corrections (FP-rate) smaller numbers are better

Figure 4 shows the average numbers of TP and FP over all four datasets, normalized to CARE 2.0. For dataset S4, CARE 1.0 used 20 hash tables and excluded quality scores. CARE 2.0 used 2-bit quality scores. These changes were necessary to reduce memory consumption. In terms of TP, all tools perform well without much of a difference. BFC achieves the greatest number of true-positive corrections, which is around two percent greater than the TPs of SGA and CARE 2.0. In contrast, the number of false-positive corrections varies significantly per tool. One can see that CARE 2.0 achieves over an order-of-magnitude smaller number of FPs which makes it superior to the other tools. Compared to CARE 1.0, the average number of FPs, $(\sum _{i=1}^{4} FP_{S_i}) / 4$, could be improved by a factor of 1.22 by using paired-end mode and a random forest. The average FP improvement factor is 1.87 ( $= (\sum _{i=1}^{4} \textit{CARE1-FP}_{S_i} / \textit{CARE2-FP}_{S_i}) / 4$). Detailed numbers of TPs and FPs for each tool and dataset are available in the supplement.

### Real-world data

#### De-novo assembly

Assembly reports for corrected and uncorrected datasets are shown in Table 2 in terms of N50 and NGA50. CARE 2.0 is able to further improve the results of CARE 1.0 for each dataset. In addition, it produces the best assembly results on datasets R1 and R3, and the second best result on dataset R2. The full QUAST reports for each dataset can be found in the supplement.

**Table 2:** A selection of assembly metrics for the real datasets

	NG50			NGA50		
	R1	R2	R3	R1	R2	R3
Uncorrected	8518	43,568	5506	6325	35,938	5439
CARE 1.0	8568	44,397	10,046	6333	36,609	9278
CARE 2.0 pe	8537	44,165	10,251	6310	36,459	9604
CARE 2.0 pe forest	**8575**	45,807	**10,624**	**6350**	37,649	**10,014**
Musket	6635	33,375	9886	4831	28,470	9083
SGA	8513	42,350	9806	6322	35,140	9099
Karect	8220	34,203	10,263	6077	29,503	9633
Bcool	8189	**50,280**	7727	5962	**40,116**	7032
Lighter	8108	33,301	10,162	5988	28,015	9300
BFC	8497	42,281	10,470	6284	34,782	9779

Bold indicates the best values per column

#### *k*-mer statistics

**Fig. 5:**
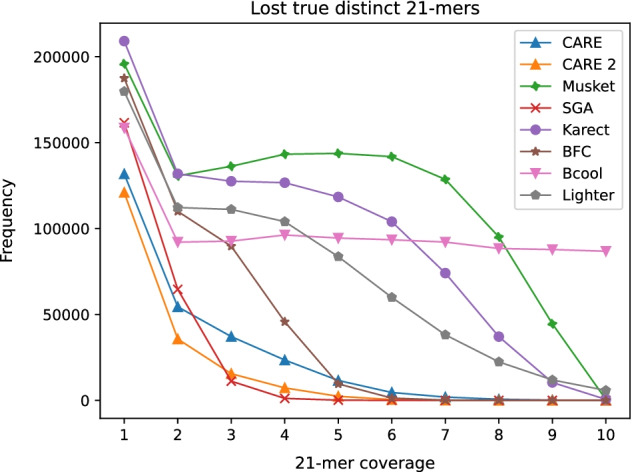
Number of low-coverage 21-mers of dataset R2 which have been falsely removed from the *k*-mer spectrum during error correction. Smaller numbers are better

**Fig. 6:**
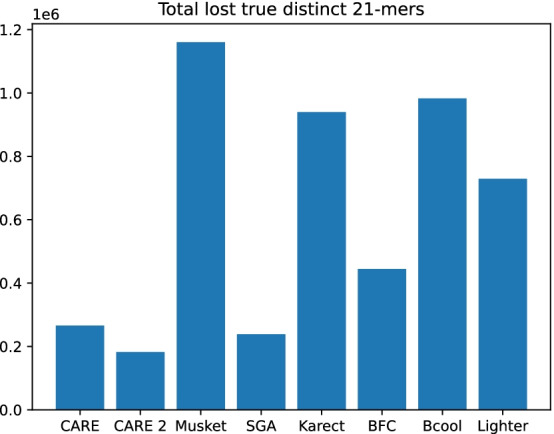
Total number of low-coverage 21-mers (coverage 1–10) of dataset R2 which have been falsely removed from the *k*-mer spectrum during error correction. Smaller numbers are better

**Fig. 7:**
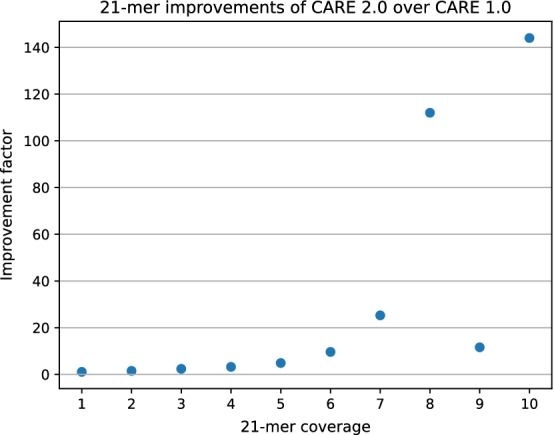
Improvement of CARE 2.0 over CARE 1.0 in terms of lost 21-mers

To quantify false-positive corrections on real-world datasets where the location of errors is generally unknown, *k*-mer spectra have been inspected for uncorrected reads, corrected reads, and the corresponding reference genome. A *k*-mer is called *true* *k*-mer if it occurs in the reference genome. During error correction true *k*-mers which are present in the uncorrected reads should not be altered to keep the correct genome information. *k*-mers which are present in both the uncorrected reads and the genome, but are missing from the corrected reads are called *lost true* *k*-mers. A perfect error correction algorithm should not introduce lost true *k*-mers. Figure 5 displays the number of lost true distinct 21-mers of dataset R2 for different error correction tools. Low-coverage *k*-mers can be easily lost during error correction because there is not much supporting information. For example, a *k*-mer could be changed into a more frequent one. With its high precision corrections CARE 2.0 is able to keep the greatest number of low-coverage true 21-mers. The total number of low-coverage lost 21-mers in dataset R2 is presented in Fig. 6. Figure 7 shows a comparison between results of CARE 2.0 and CARE 1.0. CARE 2.0 significantly reduces the number of lost 21-mers with a coverage of 5–10. The exact numbers of lost true 21-mers for each tool and dataset are available in the supplement.

### Runtime and memory consumption

Benchmarks have been performed on a Linux workstation with an AMD Ryzen Threadripper 3990X 64-core processor and 256 GB system memory. Additionally, the workstation comprises of a NVIDIA Geforce RTX 3090 GPU with 24 GB memory which will be used for the GPU-implementation of CARE. Runtime and memory consumption have been measured using the unix command /usr/bin/time -v. Results for simulated human dataset S4 are shown in Table 3. Benchmarks of CARE, except for CARE 2.0 PE Forest* have been performed using 20 hash tables and with quality scores disabled. CARE 2.0 PE Forest* instead shows the time using 48 hash tables, with enabled quality scores. To reduce memory consumption, a lossy 2-bit compression of quality scores has been used. The GPU programs have been run with a reduced number of threads because only a limited number of threads can be reasonably utilized. 128 threads (64 + hyper-threading) have been used for Karect instead of 64 to further reduce the runtime, which was known by previous benchmarks to be significantly greater than the time of other tools.

**Table 3:** Resource usage of error correction tools for the correction of Human dataset S4

Name	#threads	Runtime	Memory
CARE 1.0 (CPU)	64	157	241
CARE 1.0 (GPU)	16	84	238
CARE 2.0 SE (CPU)	64	95	234
CARE 2.0 SE (GPU)	16	42	220
CARE 2.0 PE (CPU)	64	98	235
CARE 2.0 PE (GPU)	16	43	220
CARE 2.0 PE Forest (CPU)	64	120	234
CARE 2.0 PE Forest (GPU)	16	60	221
CARE 2.0 PE Forest* (CPU)	64	265	245
CARE 2.0 PE Forest* (GPU)	16	180	245
Musket	64	249	138
SGA	64	334	37
Karect	128	6209	240
Bcool	64	346	43
Lighter	64	39	16
BFC	64	85	108

The runtime is given in minutes. Memory consumption is given in GB

First, we compare different runs of CARE. CARE 2.0 introduces a number of optimizations which reduce the runtime by up to a factor of 2. The paired-end mode does not introduce significant algorithmic complexity which results in a runtime similar to the single-end mode. Error correction using a random forest has a negative impact on performance. The main contributor to the runtime is the high number of random memory accesses when traversing the trees. CARE 2.0 PE Forest* uses more hash tables and (compressed) quality scores. The quality scores do not fit into GPU memory and thus need to be accessed from CPU memory via slow PCI-e interconnect. Compared to other error correctors, CARE 2.0 in general is able to match the performance of the *k*-mer based tools Lighter and BFC. Using the best corrections settings, which increase the runtime, CARE 2.0 can still be considered one of the faster tools.

In terms of reported memory usage, CARE has the highest memory consumption. However, in general the measured memory is not strictly required. At its core, CARE needs the reads and the hash tables to be located directly in memory. The remaining free memory, if any, is then utilized to store temporary results. On systems with less memory this leads to results being stored on disk instead at a cost of performance. A similar principle applies to GPU memory. GPU versions of CARE occupy close to the maximum of available GPU memory, which is 24 GB. They attempt to cache as much read data as possible on the GPU for fast access. The remaining read data has to be fetched from system memory.

## Discussion

The ultimate goal of read error correction is to produce a dataset of error-free reads given a dataset of possibly erroneous reads. This is achieved by finding and removing all existing errors, without introducing new errors. To date, no such perfect algorithm exists. However, current state-of-the-art algorithms for Illumina datasets are able to correct the vast majority of substitution errors, at the expense of relatively few false-positive corrections. In our evaluation on simulated datasets, excluding S4, we observed that around $98\%$ of all errors could be corrected while around $0.5\%$ of performed corrections are wrong. The exact numbers are tool-dependent and dataset-dependent and can be found in the supplement. The issue with error correction algorithms lies in the absolute number of false-positives. Absolute numbers can still reach hundreds of thousands on medium-sized datasets, and tens of millions on large datasets. Thus, even with a great amount of errors corrected, erroneous reads resulting from false-positive corrections can still affect analysis in a negative way. With CARE 2.0, we are able to further reduce the absolute FP numbers. At the same time, our correct modifications of reads are on par with those of other programs.

Simulated datasets are a simple way to assess the potential of an error corrector. However, good results on simulated data may not necessarily translate to good results on real-world datasets since error models of simulators could be different compared to that of sequencing machines. We have verified that good results of CARE 2.0 can be observed for both simulated datasets and real-world datasets. Evaluation with real reads can be challenging because usually corresponding error-free reads are not available to count corrected errors. Instead, we have used publicly available reference genomes to evaluate the impact of real-world error correction on the *k*-mer spectra as well as on de-novo assembly. The quality of both the resulting *k*-mer spectrum and the assembled contigs is susceptible to sequencing errors.

The impact of wrong corrections on the *k*-mers is two-fold. On one hand, a valid *k*-mer could be changed into a one which does not appear in the reference genome. On the other hand, *k*-mers of reads spanning low-coverage regions could be altered into more frequent ones. While this does not introduce wrong information to the spectrum, correct information is removed instead. With its low number of false-positive corrections the algorithm of CARE 2.0 removes the fewest correct 21-mers from the dataset compared to other tools.

Similar arguments can be made for de-novo assembly. When a *k*-mer is falsely changed into a different existing *k*-mer the corresponding genome locations, which could have a large distance in the genome, may appear to be in close proximity because they are connected through this *k*-mer. Losing *k*-mers in low-coverage regions through wrong corrections can prevent the assembly of this region altogether because it may not be possible to find overlapping *k*-mers between reads of that region. These observations also apply to false-negative corrections, i.e. errors which remain undetected or uncorrected. Thus, both a high number of TP, and small number of FP are desired.

Our presented evaluation of CARE 2.0 using real-world datasets focuses on *k*-mer analysis and de-novo genome assembly. Previous work has also investigated the impact of error correction on other types of downstream analysis, such as variant calling [2]. The results indicate that error correction can increase the number of correctly called SNPs and decrease the number of false positive calls. We thus plan to investigate the applicability of CARE 2.0 in different downstream analysis pipelines as part of our future work.

## Conclusions

NGS datasets are affected by errors. While there exists a variety of tools which tackle the problem of error correction in preparation of downstream analysis, its results can be negatively impacted by the presence of false-positive corrections. We have revised the algorithm of CARE, an MSA-based tool for error correction of Illumina datasets, and extended it to utilize random forests to make decisions regarding the correction of individual nucleotides. These changes combined have resulted in increased sensitivity, specificity, as well as program efficiency.

In the future, further research can be conducted in two areas. On one hand, we may try to adapt our algorithm to target other sequencing technologies such as Oxford Nanopore or PacBio which are long-read platforms. This will introduce new challenges due to significantly longer reads, different types of sequencing errors like insertions and deletions, and higher error-rates. For instance, the current alignment computation via shifted hamming distance will no longer be viable. It would need to be replaced by an actual semi-global alignment. On the other hand, we have shown that the use of a random forest improves correction quality. Other machine learning techniques may also prove beneficial. For example, our MSAs could be interpreted as a multi-channel image which can be passed to a deep neural network for the detection of sequencing errors. Our hand-selected features which are currently used for the random forest may not be optimal whereas a neural network could learn different, better features from the MSAs.

## Supplementary information

**Additional file 1.** Program arguments and detailed results.

## Data Availability

Instructions to generate simulated datasets are included in the Additional file 1. The real datasets are publicly available at NCBI SRA using the accession numbers SRR543736, SRR988075, and SRR067577. **Availability and requirements** Project name: CARE 2.0. Project home page: https://github.com/fkallen/CARE. Operating system(s): Linux. Programming language: C++, CUDA. Other requirements: The GPU version requires CUDA toolkit ≥ 11. License: GPLv3. Any restrictions to use by non-academics: No additional restrictions.

## Abbreviations

NGS: Next-generation sequencing
MSA: Multiple sequence alignment
TP: True-positive
FP: False-positive
