# Supplementary material for: CARE 2.0: reducing false-positive sequencing error corrections using machine learning
Source: BMC Bioinformatics. 2022 Jun 13;23:227. doi: 10.1186/s12859-022-04754-3 (PMC9195321; doi:10.1186/s12859-022-04754-3)
Supplement: Supplementary file 1 — Additional file 1. Program arguments and detailed results. [file 12859_2022_4754_MOESM1_ESM.pdf]

CARE 2.0: Reducing false-positive sequencing error corrections using machine learning

Supplementary file

Table of Contents

A Command lines for error correctors....2
B Command lines for evaluation....3
C Preparation and evaluation of simulated datasets....4
D Simulated results....5
E Assemblyresults....6
F K-mer results....9

A Command lines for error correctors

SGA

- $SGA preprocess --pe-mode 2 --permute-ambiguous --no-primer-check -m 1 -o $outputdirectory/sga_temp $fastqreads
- $SGA index --no-reverse -a ropebwt -t $threads $outputdirectory/sga_temp
- $SGA correct -k 41 -t $threads -o $outputdirectory/sga.fastq $outputdirectory/sga_temp

Karect

- $KARECT -correct -inputfile=$fastqreads -tempdir=$outputdirectory -celltype=diploid -resultprefix="karect_" -matchtype=hamming -resultdir=$outputdirectory -kmer=9 -memory=$memory -threads=$threads

Musket

- $MUSKET -inorder -p $threads -o $outputdirectory/musket.fastq $fastqreads

BCOOL

- $BCOOL -u $fastareads -o $outputdirectory/bcool -t $threads

bfc

- $BFC -s $approxgenomesize -k 27 -t $threads $fastqreads > $outputdirectory/bfc.fastq

Lighter

- $LIGHTER -K 20 $approxgenomesize -t $threads -r $fastqreads -od $outputdirectory/lighter

CARE 1.0

- $CARE -i $fastqreads -c $coverage -o care1.fastq -d . --excludeAmbiguous -m $memory -p -t $threads --candidateCorrection -g 0 -k 20 -h 48 -q

CARE 2.0 PE

- $CARE -i $fastqreads -c $coverage -o care1.fastq -d . --excludeAmbiguous -m $memory -p -t $threads --candidateCorrection -g 0 -k 20 -h 48 -q --pairmode PE --qualityScoreBits 8

CARE 2.0 PE Forest with 2-bit quality scores

- $CARE -i $fastqreads -c $coverage -o care1.fastq -d . --excludeAmbiguous -m $memory -p -t $threads --candidateCorrection -g 0 -k 20 -h 48 -q --pairmode PE --correctionType 1 --ml-forestfile anchorforest.rf --correctionTypeCands 1 --ml-cands-forestfile candidateforest.rf --thresholdAnchor 93 --thresholdCands 15 --maxForestTreesAnchor 128 --maxForestTreesCands 128 --qualityScoreBits 2

B Command lines for evaluation

SPAdes

- spades.py -t $threads -m $memory --only-assembler --12 $readsfile -o spadesout

QUAST

- quast.py spadesout/contigs.fasta -R $genome -o quastout --plots-format ps --labels “program name” --threads $threads

To create the k-mer statistics we analyzed the output of Jellyfish with custom scripts which are included in the CARE repository

#Run once per uncorrected dataset
jellyfish count -m 21 -C -s 100M -t 16 uncorrectedreads
jellyfish dump mer_counts.jf > uncorrectedkmers.fasta

./findkmersingenome uncorrectedkmers.fasta 21 genomefile uncorrectedkmersInGenome.fasta

#Run once per corrected dataset per tool
jellyfish count -m 21 -C -s 100M -t 16 tool_correctedreads
jellyfish dump mer_counts.jf > tool_correctedkmers.fasta

./findmissingkmers uncorrectedkmersInGenome.fasta tool_correctedkmers.fasta 21 10

C Preparation and evaluation of simulated datasets

Simulated datasets have been generated using the ART read simulator. To collect per-nucleotide correction statistics a custom program has been used. This tool is included in the CARE repository.

ART:

Simulated HiSeq2000 datasets have been generated via

```
    art_illumina -ef -f $coverage -i genome.fa -l 100 -na -o ./output -ss HS20 -
p -m 500 -s 10
```

In all cases the error-free / perfect reads have been extracted from the .sam file via SAMtools.

```
    samtools fastq output_errFree.sam > output_errFree.fq
```

Error metrics:

```
    ./arteval originalReads perfectReads correctedReads [correctedReads...]
```

D Simulated results

Results for S1 (C.elegans)

	CARE 1.0	CARE 2.0 PE RF	BFC	Musket	SGA	BCOOL	Lighter	Karect
TP	27,730,616	28,274,622	28,719,759	27,359,612	28,088,449	28,076,865	27,759,329	28,517,942
FP	9,313	4,644	164,187	304,402	163,617	143,866	405,593	82,652
FN	1,398,422	854,416	409,279	1,769,426	1,040,589	1,052,173	1,369,709	611,096
TN	2,979,432,349	2,979,437,318	2,979,277,775	2,979,137,560	2,979,278,345	2,979,298,096	2,979,036,369	2,979,359,310
Fp / 1M cor	335.73	164.22	5,684.37	11,003.54	5,791.33	5,097.88	14,400.64	2,889.87
Sensitivity	0.95	0.97	0.99	0.94	0.96	0.96	0.95	0.98
Specificity	1.00	1.00	1.00	1.00	1.00	1.00	1.00	1.00
Precision	1.00	1.00	0.99	0.99	0.99	0.99	0.99	1.00

Results for S2 (D.melanogaster)

	CARE 1.0	CARE 2.0 PE RF	BFC	Musket	SGA	BCOOL	Lighter	Karect
TP	33,605,031	33,959,878	34,570,352	33,908,707	33,705,222	34,029,313	33,985,269	34,446,466
FP	5,249	2,129	51,866	102,655	42,008	46,499	124,522	49,202
FN	1,330,793	975,946	365,472	1,027,117	1,230,602	906,511	950,555	489,358
TN	3,573,675,327	3,573,678,447	3,573,628,710	3,573,577,921	3,573,638,568	3,573,634,077	3,573,556,054	3,573,631,374
Fp / 1M cor	156.17	62.69	1,498.06	3,018.26	1,244.78	1,364.57	3,650.62	1,426.32
Sensitivity	0.96	0.97	0.99	0.97	0.96	0.97	0.97	0.99
Specificity	1.00	1.00	1.00	1.00	1.00	1.00	1.00	1.00
Precision	1.00	1.00	1.00	1.00	1.00	1.00	1.00	1.00

Results for S3 (Human Chr.14)

	CARE 1.0	CARE 2.0 PE RF	BFC	Musket	SGA	BCOOL	Lighter	Karect
TP	23,380,267	24,477,496	24,748,136	22,511,643	24,064,241	23,819,893	23,152,988	23,936,125
FP	13,589	7,641	377,975	493,773	597,770	627,532	888,141	159,309
FN	2,271,360	1,174,131	903,491	3,139,984	1,587,386	1,831,734	2,498,639	1,715,502
TN	2,622,996,184	2,623,002,132	2,622,631,798	2,622,516,000	2,622,412,003	2,622,382,241	2,622,121,632	2,622,850,464
Fp / 1M cor	580.88	312.07	15,043.12	21,463.34	24,238.49	25,668.63	36,942.57	6,611.58
Sensitivity	0.91	0.95	0.96	0.88	0.94	0.93	0.90	0.93
Specificity	1.00	1.00	1.00	1.00	1.00	1.00	1.00	1.00
Precision	1.00	1.00	0.98	0.98	0.98	0.97	0.96	0.99

Result for S4 (Human)

	CARE 1.0 (no quality scores, H = 20)	CARE 2.0 Forest PE (2-bit q-scores, H = 48)	BFC	Musket	SGA	BCOOL	Lighter	Karect
TP	701,159,634	801,427,728	814,505,200	647,616,649	776,653,317	768,702,333	696,164,863	725,547,371
FP	1,457,191	1,240,994	16,973,353	34,635,118	34,127,165	39,623,863	55,703,403	3,895,037
FN	184,563,944	84,295,850	71,218,378	238,106,929	109,070,261	117,019,997	189,558,715	160,176,207
TN	90,579,493,431	90,579,709,628	90,563,977,269	90,546,315,504	90,546,823,457	90,541,199,507	90,525,247,219	90,577,055,585
Fp / 1M cor	2,073.95	1,546.08	20,413.46	50,765.89	42,091.74	49,019.64	74,086.65	5,339.75
Sensitivity	0.79	0.90	0.92	0.73	0.88	0.87	0.79	0.82
Specificity	1.00	1.00	1.00	1.00	1.00	1.00	1.00	1.00
Precision	1.00	1.00	0.98	0.95	0.96	0.95	0.93	0.99

E Assemblyresults

QUAST output for dataset R1 (C.elegans)

Assembly	Uncorrected	CARE 1.0	CARE 2.0 PE RF	BFC	Musket	SGA	BCOOL	Lighter	Karect
# contigs (>= 0 bp)	91061	89866	89528	90469	97546	90334	92332	91731	93538
# contigs (>= 1000 bp)	20132	20079	20076	20160	22830	20111	20464	20608	20505
# contigs (>= 5000 bp)	6201	6182	6205	6207	6036	6195	6161	6234	6205
# contigs (>= 10000 bp)	2336	2334	2329	2338	1850	2326	2287	2283	2312
# contigs (>= 25000 bp)	280	287	280	292	184	283	282	266	298
# contigs (>= 50000 bp)	65	65	63	59	42	64	65	54	55
Total length (>= 0 bp)	116361901	116228171	116220444	116135208	115401704	116252369	116087282	115826839	115943548
Total length (>= 1000 bp)	103912795	103861149	103864431	103596162	101068763	103835902	103327923	102951858	103117977
Total length (>= 5000 bp)	70663890	70671920	70762273	70457430	61558228	70619925	69194703	68807877	69134950
Total length (>= 10000 bp)	43599890	43694725	43551819	43361927	32357653	43498032	42114184	41083313	41739197
Total length (>= 25000 bp)	13566746	13788334	13603742	13498816	8683702	13634663	13050583	11711161	12494616
Total length (>= 50000 bp)	6657935	6659904	6569238	5806887	3886069	6536168	5941165	4873109	4662738
# contigs	26592	26559	26573	26787	31048	26602	27124	27551	27198
Largest contig	244078	244078	244078	240394	240394	240969	240969	232406	240038
Total length	108572110	108538335	108556766	108375204	106993199	108520814	108131361	107957348	107947283
Reference length	100286070	100286070	100286070	100286070	100286070	100286070	100286070	100286070	100286070
GC (%)	38.47	38.48	38.48	38.45	38.4	38.47	38.5	38.39	38.43
Reference GC (%)	35.44	35.44	35.44	35.44	35.44	35.44	35.44	35.44	35.44
N50	7659	7688	7696	7607	6126	7660	7436	7378	7464
NG50	8518	8568	8575	8497	6635	8513	8189	8108	8220
N75	3546	3548	3545	3517	2873	3535	3435	3406	3420
NG75	4365	4363	4363	4308	3397	4337	4174	4116	4142
L50	3559	3541	3551	3579	4578	3557	3682	3789	3729
LG50	3046	3032	3042	3076	4052	3047	3178	3292	3239
L75	8760	8732	8741	8796	10963	8762	9036	9188	9074
LG75	7177	7156	7160	7234	9357	7183	7479	7648	7543
# misassemblies	1276	1265	1268	1321	2058	1273	1738	1424	1358
# misassembled contigs	1219	1208	1205	1260	1930	1217	1639	1349	1289
Misassembled contigs length	9064384	9064782	8980991	9535411	10835675	9214818	12096964	9467696	9441387
# local misassemblies	272	285	278	277	313	288	324	279	298
# scaffold gap ext. mis.	0	0	0	0	0	0	0	0	0
# scaffold gap loc. mis.	0	0	0	0	0	0	0	0	0
# unaligned mis. contigs	3	5	6	5	5	5	3	4	5
# unaligned contigs	4906 + 55 part	4897 + 55 part	4903 + 53 part	5004 + 49 part	5080 + 57 part	4902 + 52 part	4893 + 59 part	5065 + 53 part	4975 + 51 part
Unaligned length	16536845	16537750	16542066	16432096	15931377	16524035	16447241	16143138	16230083
Genome fraction (%)	91.322	91.303	91.306	91.262	90.134	91.292	90.924	91.095	91.035
Duplication ratio	1.005	1.005	1.005	1.005	1.007	1.005	1.005	1.005	1.005
# N's per 100 kbp	0	0	0	0	0	0	0	0	0
# mismatches per 100 kbp	18.31	18.02	17.92	18.77	149.95	18.83	23.48	39.08	23.95
# indels per 100 kbp	4.71	4.78	4.76	4.76	7.54	4.8	5.2	5.26	5.02
Largest alignment	54032	54032	54032	54032	41089	54032	54032	50923	54032
Total aligned length	91816340	91789947	91801972	91740266	90686458	91783889	91448148	91585678	91514140
NA50	5612	5625	5635	5591	4392	5611	5326	5380	5455
NGA50	6325	6333	6350	6284	4831	6322	5962	5988	6077
NA75	1912	1914	1914	1910	1542	1912	1817	1863	1874
NGA75	2709	2703	2704	2674	2056	2701	2524	2554	2570
LA50	5037	5021	5026	5053	6388	5036	5276	5235	5152
LGA50	4342	4330	4334	4371	5659	4345	4579	4558	4486
LA75	13074	13044	13051	13113	16396	13077	13727	13548	13403
LGA75	10363	10346	10349	10448	13579	10381	10999	10926	10801

QUAST output for R2 (D.melanogaster)

Assembly	Uncorrected	CARE 1.0	CARE 2.0 PE RF	BFC	Musket	SGA	BCOOL	Lighter	Karect
# contigs (>= 0 bp)	170491	161476	162278	176507	187741	170803	160783	186485	188635
# contigs (>= 1000 bp)	7103	7005	6881	7320	8576	7309	6445	8651	8598
# contigs (>= 5000 bp)	3782	3745	3655	3891	4408	3874	3389	4437	4405
# contigs (>= 10000 bp)	2659	2630	2595	2664	2854	2678	2503	2838	2780
# contigs (>= 25000 bp)	1336	1343	1332	1321	1217	1316	1356	1211	1192
# contigs (>= 50000 bp)	575	588	587	561	456	570	626	487	483
Total length (>= 0 bp)	136008271	135416833	135484880	136331061	137017238	136104640	134858304	137090317	137278352
Total length (>= 1000 bp)	118770197	118797868	118814150	118651913	118188444	118750809	118457277	118264198	118303600
Total length (>= 5000 bp)	110806731	111015468	111151242	110330557	107866595	110438712	111272819	107832964	107940622
Total length (>= 10000 bp)	102774604	103022192	103599606	101506200	96699657	101842764	104949720	96347313	96258975
Total length (>= 25000 bp)	81170209	81925404	82878761	79762578	70682794	79728287	86305922	70393317	70966343
Total length (>= 50000 bp)	54276219	55252123	56520262	52967381	44072081	53520966	60389701	45218837	46160245
# contigs	9227	9117	8983	9455	10748	9449	8354	10901	10811
Largest contig	535439	535437	535439	479283	579112	722059	579467	596026	579113
Total length	120260659	120279412	120291746	120153062	119729754	120251564	119801080	119860964	119874008
Reference length	120381546	120381546	120381546	120381546	120381546	120381546	120381546	120381546	120381546
GC (%)	42.42	42.42	42.42	42.42	42.44	42.42	42.44	42.44	42.44
Reference GC (%)	42.41	42.41	42.41	42.41	42.41	42.41	42.41	42.41	42.41
N50	43681	44517	45909	42343	33564	42514	50620	33426	34551
NG50	43568	44492	45807	42281	33375	42350	50280	33301	34203
N75	18624	18935	19641	17555	13423	17555	22260	13284	13253
NG75	18613	18855	19578	17414	13215	17494	21745	13004	12974
L50	700	692	663	716	843	714	617	849	816
LG50	702	693	664	719	853	716	623	856	823
L75	1753	1722	1661	1811	2260	1812	1506	2277	2227
LG75	1758	1726	1665	1821	2296	1817	1526	2306	2256
# misassemblies	809	806	818	871	800	808	844	804	790
# misassembled contigs	670	670	669	724	681	666	689	688	676
Misassembled contigs length	34791059	35808799	36419610	36649493	31246264	34950773	39732568	30226190	30509823
# local misassemblies	1048	1049	1049	1049	1026	1049	1042	1036	1026
# scaffold gap ext. mis.	0	0	0	0	0	0	0	0	0
# scaffold gap loc. mis.	0	0	0	0	0	0	0	0	0
# unaligned mis. contigs	421	424	413	415	397	426	367	414	407
# unaligned contigs	2213 + 976 part	2200 + 979 part	2198 + 986 part	2121 + 975 part	2058 + 915 part	2191 + 970 part	2247 + 924 part	2093 + 918 part	2057 + 926 part
Unaligned length	6471506	6479468	6489437	6409392	6189723	6475645	6119891	6259504	6286445
Genome fraction (%)	94.023	94.034	94.037	93.989	93.844	94.001	93.988	93.867	93.855
Duplication ratio	1.006	1.006	1.006	1.006	1.006	1.006	1.005	1.006	1.006
# N's per 100 kbp	0	0	0	0	0	0	0	0	0
# mismatches per 100 kbp	511.02	511.26	510.55	509.73	509.57	510.45	511.44	507.26	505.88
# indels per 100 kbp	106.17	106.32	106.34	106.03	105.3	106.17	106.59	105.3	105.24
Largest alignment	402892	402890	402892	335133	574421	574412	402892	436540	334373
Total aligned length	113370656	113391535	113397005	113346970	113160583	113361675	113291773	113202478	113211634
NA50	35967	36552	37672	34950	28765	35170	40281	28204	29735
NGA50	35938	36551	37649	34782	28470	35140	40116	28015	29503
NA75	15945	16295	16797	14990	11746	15170	18439	11567	11609
NGA75	15889	16248	16755	14880	11571	15107	18099	11428	11455
LA50	865	861	833	897	1014	890	782	1024	984
LGA50	866	862	834	900	1025	892	789	1033	993
LA75	2115	2088	2028	2208	2634	2185	1867	2668	2596
LGA75	2120	2093	2032	2220	2676	2191	1891	2702	2629

QUAST output for R3 (Human Chr.14)

Assembly	Uncorrected	CARE 1.0	CARE 2.0 PE RF	BFC	Musket	SGA	BCOOL	Lighter	Karect
# contigs (>= 0 bp)	412807	53159	54963	58475	55902	55798	67852	53921	58228
# contigs (>= 1000 bp)	15216	9864	9411	9472	9906	9933	12003	9764	9550
# contigs (>= 5000 bp)	5457	5099	5050	5081	5146	5147	5336	5083	5091
# contigs (>= 10000 bp)	2048	2772	2791	2759	2743	2719	2540	2752	2762
# contigs (>= 25000 bp)	165	559	623	613	551	558	363	581	594
# contigs (>= 50000 bp)	3	44	63	57	52	43	24	48	53
Total length (>= 0 bp)	111701111	88027055	88332122	88487903	88069598	88243184	88559169	88064964	88335543
Total length (>= 1000 bp)	81314165	83209317	83447184	83359018	83115505	83209578	82259060	83249019	83244550
Total length (>= 5000 bp)	56548179	70584212	71876796	71680616	70503537	70485921	65041979	70837038	71357062
Total length (>= 10000 bp)	32454389	53892959	55687408	54993442	53289730	53054605	45080093	54119026	54574474
Total length (>= 25000 bp)	5145723	19646871	22193573	21839378	19431973	19564747	12157197	20412088	20958856
Total length (>= 50000 bp)	174274	2807279	3831068	3462247	3123826	2717090	1361777	2957706	3299738
# contigs	18340	11228	10635	10697	11211	11298	13872	11056	10820
Largest contig	68123	96787	97251	99187	91213	89937	69475	91656	115425
Total length	83591797	84200372	84341485	84249145	84065329	84203410	83631792	84187081	84167916
Reference length	107349540	107349540	107349540	107349540	107349540	107349540	107349540	107349540	107349540
GC (%)	40.66	40.71	40.72	40.71	40.71	40.71	40.68	40.71	40.71
Reference GC (%)	40.89	40.89	40.89	40.89	40.89	40.89	40.89	40.89	40.89
N50	7859	14033	14749	14673	13742	13765	10925	14268	14436
NG50	5506	10046	10624	10470	9886	9806	7727	10162	10263
N75	4000	7167	7614	7522	7144	7069	5561	7275	7447
NG75	1167	2052	2231	2185	2025	2056	1494	2089	2135
L50	3104	1777	1678	1692	1785	1785	2229	1743	1720
LG50	4910	2751	2596	2630	2782	2782	3518	2708	2674
L75	6828	3861	3657	3699	3900	3915	4897	3808	3752
LG75	14473	8065	7542	7634	8148	8126	10593	7949	7759
# misassemblies	135	773	582	638	787	823	1142	836	604
# misassembled contigs	135	719	554	603	734	763	1066	756	577
Misassembled contigs length	1048587	10096064	8659050	9331567	10359789	10245459	11683533	10692919	8773199
# local misassemblies	41	53	44	43	39	44	53	44	35
# scaffold gap ext. mis.	0	0	0	0	0	0	0	0	0
# scaffold gap loc. mis.	0	0	0	0	0	0	0	0	0
# unaligned mis. contigs	0	1	0	0	0	0	0	0	0
# unaligned contigs	4 + 1 part	7 + 3 part	7 + 2 part	6 + 5 part	3 + 3 part	9 + 1 part	2 + 11 part	4 + 8 part	6 + 2 part
Unaligned length	3392	7479	6075	7778	3742	6870	15254	12612	6365
Genome fraction (%)	94.122	94.959	95.15	95.064	94.854	94.946	94.141	94.947	94.992
Duplication ratio	1.006	1.004	1.004	1.004	1.004	1.004	1.006	1.004	1.003
# N's per 100 kbp	0	0	0	0	0	0	0	0	0
# mismatches per 100 kbp	99.87	109.36	106.19	106.52	112.58	108.32	123.33	109.32	105.09
# indels per 100 kbp	18.4	19.47	19.38	19.31	19.52	19.21	19.35	19.79	19.05
Largest alignment	68123	96787	97251	93237	84681	89937	69411	90877	115425
Total aligned length	83364584	83960442	84111397	84043801	83857251	83964150	83323458	83969754	83977716
NA50	7785	13089	13847	13621	12866	12783	10075	13153	13621
NGA50	5439	9278	10014	9779	9083	9099	7032	9300	9633
NA75	3946	6601	7110	7005	6533	6435	5012	6650	6948
NGA75	1107	1844	2025	1988	1802	1830	1315	1856	1936
LA50	3126	1877	1766	1782	1889	1892	2387	1858	1802
LGA50	4953	2929	2743	2782	2966	2967	3794	2907	2815
LA75	6896	4135	3873	3922	4175	4198	5312	4105	3963
LGA75	14741	8730	8056	8186	8823	8832	11663	8654	8279

F K-mer results

Lost true distinct 21-mers in R1

coverage	CARE 1.0	CARE 2.0 PE RF	BFC	Musket	SGA	BCOOL	Lighter	Karect
1	3,596	2,888	11,496	141,236	5,567	6,675	44,807	17,682
2	342	247	4,710	110,990	1,309	3,045	23,429	7,092
3	79	39	2,928	94,440	135	1,902	15,917	4,764
4	50	9	845	84,262	25	1,449	10,703	3,265
5	14	2	172	74,006	3	1,368	6,985	2,298
6	17	1	41	60,491	3	1,387	4,495	1,582
7	25	0	29	42,896	0	1,237	2,476	919
8	7	0	4	19,948	2	1,307	1,305	544
9	11	0	1	476	0	1,447	644	203
10	21	0	1	83	0	1,686	294	48
Sum	4,162	3,186	20,227	628,828	7,044	21,503	111,055	38,397

Lost true distinct 21-mers in R2

coverage	CARE 1.0	CARE 2.0 PE RF	BFC	Musket	SGA	BCOOL	Lighter	Karect
1	131,778	121,004	187,469	195,864	161,570	158,568	179,773	209,077
2	54,453	35,734	110,031	130,634	64,626	92,080	112,228	131,845
3	37,141	15,523	89,729	136,256	11,182	92,686	111,140	127,533
4	23,497	7,275	45,748	143,262	1,167	96,251	104,020	126,690
5	11,599	2,377	9,599	143,747	133	94,475	83,661	118,392
6	4,595	477	1,353	141,857	46	93,473	59,933	104,004
7	1,921	76	175	128,612	12	92,133	38,153	74,085
8	672	6	90	95,111	9	88,405	22,421	37,102
9	174	15	44	44,584	8	87,783	11,898	10,431
10	144	0	21	574	1	86,811	5,825	599
Sum	265,974	182,487	444,259	1,160,501	238,754	982,665	729,052	939,758

Lost true distinct 21-mers in R3

coverage	CARE 1.0	CARE 2.0 PE RF	BFC	Musket	SGA	BCOOL	Lighter	Karect
1	21,665	20,072	29,126	36,154	23,732	21,967	31,698	34,237
2	2,589	2,369	7,164	12,853	4,391	4,197	8,647	9,076
3	551	537	3,939	9,793	926	2,219	5,529	5,471
4	178	162	1,996	9,541	241	2,257	4,265	4,486
5	93	68	636	10,397	84	3,199	3,622	3,956
6	28	23	141	9,059	42	5,439	2,539	2,952
7	11	25	50	4,773	30	7,679	1,456	1,157
8	1	4	21	81	5	10,763	769	284
9	1	3	4	13	3	14,377	349	108
10	0	2	4	3	1	18,434	109	64
Sum	25,117	23,265	43,081	92,667	29,455	90,531	58,983	61,791
